# Level of Healthcare Facility and Psychosocial Factors Influence Perceived Self-Efficacy for Appropriate Use of Hydroxyurea: Experience from Caregivers of Children with Sickle Cell Disease in Tanzania

**DOI:** 10.3390/healthcare13131500

**Published:** 2025-06-24

**Authors:** Mwashungi Ally, Deodatus Kakoko, Tone Kristin Omsland, Calvin Swai, Emmy Metta, Kåre Moen, Elia John Mmbaga, Melkizedeck Leshabari, Mbonea Yonazi, Agnes Jonathan, Julie Makani, Emmanuel Balandya

**Affiliations:** 1 Sickle Cell Program, Department of Hematology and Blood Transfusion, Muhimbili University of Health and Allied Sciences, 9-United Nations Road, Upanga, Dar-es-Salaam 11103, Tanzania; myonazi@gmail.com (M.Y.); ajonathan@blood.ac.tz (A.J.); jmakani@blood.ac.tz (J.M.); ebalandya@yahoo.com (E.B.); 2 Department of Behavioral Sciences, Muhimbili University of Health and Allied Sciences, Dar-es-Salaam 11103, Tanzania; deodatuskakoko@gmail.com (D.K.); emetta2000@gmail.com (E.M.); mleshabari@gmail.com (M.L.); 3 Department of Community Medicine and Global Health, University of Oslo, 0450 Oslo, Norway; t.k.omsland@medisin.uio.no (T.K.O.); kare.moen@medisin.uio.no (K.M.); elia.mmbaga@medisin.uio.no (E.J.M.); 4 Department of Educational Psychology and Curriculum Studies, The University of Dodoma, Dodoma 41218, Tanzania; czakaria@ualberta.ca; 5 Department of Hematology and Blood Transfusion, Muhimbili National Hospital, Dar-es-Salaam 65000, Tanzania

**Keywords:** social support, depressive symptoms, self-efficacy for appropriate medication use, hydroxyurea, sickle cell disease

## Abstract

**Background**: Sickle cell disease (SCD) is associated with high physical and psychosocial burden among patients and their families. Hydroxyurea (HU) improves health-related quality of life by preventing SCD complications. Despite its availability, HU is underutilised in Tanzania. Perceived self-efficacy for appropriate medication use influences medication usage among individuals with chronic illnesses. We studied factors associated with caregivers’ perceived self-efficacy for appropriate use of HU and its association with HU usage among children with SCD in Dar-es-Salaam. **Methods**: We conducted a cross-sectional study from May to August 2023. We enrolled 374 caregivers of children with SCD from two regional and two national hospitals. We adapted the self-efficacy for appropriate medication use scale, a multidimensional perceived social support scale, and a patient health questionnaire for assessment of self-efficacy, social support, and depressive symptoms, respectively. **Results**: Three-quarters of caregivers had high perceived self-efficacy scores for medication use. Attending national hospitals, high social support, and absence of depressive symptoms were positively associated with perceived self-efficacy (adjusted beta coefficient aβ 2.3, 95% CI 0.5–4.2; aβ 9, 95% CI 7.1–10.9; and aβ 5.3, 95% CI 2.8–7.8, respectively). Caregivers with high self-efficacy were 5.3 times more likely to give HU to their children compared with those with low self-efficacy (incidence rate ratio 5.3, 95% CI 3.3–8.3). **Conclusions**: Hospital levels and psychosocial factors influence caregivers’ perceived self-efficacy for appropriate HU use. We recommend targeted interventions to enhance psychosocial support among caregivers to increase caregivers’ perceived self-efficacy and HU utilization among children with SCD in Tanzania.

## 1. Introduction

Sickle cell disease (SCD) is a life-threatening inherited blood disease characterized by chronic anemia and recurrent pain [1,2]. Approximately 300,000 to 400,000 babies are born with SCD every year, with over three-quarters of these living in Sub-Saharan Africa (SSA) [3,4]. Tanzania ranks fifth among countries with the highest prevalence of SCD globally with approximately 11,000 to 14,000 live births of babies with SCD [5,6,7]. The prevalence of SCD trait (heterozygous S; HbAS) ranges between 13% to 20%, and the prevalence of SCD (homozygous S; HbSS) ranges between 0.8–1.4% [4,5,8]. Being a chronic disease, SCD is associated with high psychosocial burden to patients and their families [1,4,9,10]. Hydroxyurea (HU) is a disease-modifying medication that, when used properly, reduces the incidence of SCD complications and improve patients’ survival and health-related quality of life [11,12,13,14,15]. Despite the ongoing efforts to improve its availability and affordability, such as inclusion of HU in the national SCD treatment guidelines, availability of HU in the National Medical Stores department, removal of restrictions in HU coverage among the National Health Insurance Fund (NHIF) coverage, and HU coverage by private health insurance schemes, HU remains significantly underutilized in SSA, with only a quarter of patients with SCD being reported to use HU in Tanzania [4,16,17,18].

Perceived self-efficacy, as defined by Bandura, is a strong belief in one’s ability to execute behaviors necessary to accomplish a certain task [19]. Self-efficacy for appropriate use of medications refers to an individual’s ability to use medications as prescribed [20]. Individual’s perceived self-efficacy for appropriate use of medications is an important construct in studying optimal use of medications and one of the most potent determinants of behavior change in disease management [21,22]. Studies have shown that high perceived self-efficacy is positively associated with optimal use of medications in patients with chronic diseases and better health outcomes [23,24,25].

Self-efficacy for appropriate use of medication is influenced by various factors such as experiences, knowledge of the disease and prescribed medications (mastery experience), social support from family, friends, significant others, healthcare providers, and support groups (vicarious experience and verbal persuasion), as well as the physiological and emotional well-being of the patients and their caregivers [21,26,27,28]. Social support from family, friends, and support groups positively influences self-efficacy by providing emotional encouragement and acting as a source of information, knowledge, and reminders. Support from healthcare providers, including provider–caregiver interactions, empathy, respect for cultural context, and communication clarity, impact caregiver trust, treatment understanding, high-quality decision-making, and self-efficacy [29,30,31]. This strengthens an individual’s confidence in managing the prescribed medication routines, enhancing adherence and leading to improved outcomes [26,29,32]. On the other hand, studies among underserved families in low- and middle-income countries have shown that caregivers with limited social support, such as single parents or those caring for multiple children, often face logistical challenges like missing work and loss of income due to the time spent caring for their sick children [33]. This compromises their ability to adhere to clinics schedules or refill prescriptions on time. Moreover, lack of provider support such as provider biases, poor communication, or perceived judgement can diminish caregiver confidence, especially in the management of patients with chronic diseases such as SCD, where caregivers have high psychosocial burden [30]. Positive emotional well-being enhances self-efficacy, whereas anxiety and depression negatively influence self-efficacy by impairing motivation to use medications, enhancing negative health beliefs, and undermining individual’s confidence in the ability to effectively manage medication routines [34]. Addressing depressive symptoms and social support through targeted interventions is associated with improved self-efficacy and medication usage [32,34].

The existing literature from Tanzania has shown the role of caregiver and patient’s self-efficacy in the management of chronic illnesses such as HIV, indicating that higher self-efficacy is associated with improved medication-taking behaviors and health outcomes [35]. Moreover, studies conducted in SSA and other countries assessing perceived self-efficacy in various aspects of management of SCD among caregivers and patients with SCD have shown that self-efficacy is associated with health literacy, transition readiness, self-management, and health-related quality of life [18,36,37,38,39,40]. However, there is limited research on caregivers’ perceived self-efficacy for the appropriate use of HU in the management of children with SCD. These findings underscore the importance of examining caregiver self-efficacy in the Tanzanian context to inform targeted interventions that can improve caregivers’ confidence in their ability to use HU and improve HU usage. In this study, we assessed factors associated with caregivers’ perceived self-efficacy for appropriate use of HU and the relationship between caregivers’ perceived self-efficacy and the use of HU in the management of their children with SCD in Dar es Salaam, Tanzania.

## 2. Materials and Methods

### 2.1. Study Design

We conducted a hospital-based cross-sectional study using quantitative methods in Dar es Salaam, Tanzania, from 1 May 2023 to 31 August 2023.

### 2.2. Study Setting

The study was conducted in four public secondary (regional) and tertiary (national) referral hospitals in the Dar es Salaam region in Tanzania. Dar es Salaam is the fifth-largest city in Africa and the biggest city in Tanzania, with more than 5 million residents. There are 572 registered health facilities in the region, and among them 42 are hospitals, 48 health centers, and 423 dispensaries. Public health facilities account for 19% of all facilities [41]. However, due to limited access to specialized pediatric hematology care caused by a shortage of hematologists, SCD-related services, and dedicated SCD clinics, patients with SCD attend regular follow-up clinics at dedicated SCD clinics in regional hospitals and national hospitals based on their physical addresses and the severity of their SCD complications [4]. There are 3 regional referral hospitals (Amana, Temeke, and Mwananyamala) and 1 national hospital with 2 campuses, i.e., Muhimbili National Hospital (MNH) Upanga and Mloganzila. These hospitals are found in urban areas in Dar es Salaam with relatively better infrastructure and accessibility. MNH also receives patients from all regions who have acquired major SCD complications and require super-specialized services. All dedicated SCD clinics in Dar es Salaam are clinical sites for the Sickle Pan-African Research Consortium (SPARCO)-Tanzania. SCD patients at these hospitals are registered in a REDCap-based electronic database managed by SPARCO-Tanzania, allowing for proper recording of information at each follow-up visit. These sites offer comprehensive SCD care including screening and diagnosis of SCD, provision of routine SCD medications such as folic acid, hydroxyurea, and penicillin V, health education, as well as screening, prevention, and management of acute and chronic SCD complications. Additionally, newborn screening for SCD is available at Amana and Temeke RRH. We excluded one regional hospital (Mwananyamala) because HU was not available at the hospital during the study period. Exclusion of Mwananyamala RRH allowed us to study the effect of self-efficacy on HU use while controlling for unavailability of HU which is one of the known major barriers to HU use in Tanzania and SSA [4,18,42]. By the year 2021, about 81% of patients registered in the SPARCO-Tanzania database were children aged below 18 years, and 12% were on HU [17].

### 2.3. Sample Size Calculation

The formula for calculation of the sample size for a finite population was used to obtain the minimum sample size for the study. In the formula, *n* = *n*_0_/1 + [(*n_0_* − 1)/N], where *n*_0_ = z^2^ (1 − P)/e^2^. Since the proportion of caregivers with high self-efficacy for appropriate use of HU was unknown, we used a prevalence (P) of 50%, Z score of 1.96, standard error of 0.05, and population (N) of 1829. The minimum estimated sample size required to attain a power of 80% was 318. To strengthen validity of our study, we performed a post hoc power analysis using an observed prevalence of 75% and a sample size of 374. The analysis yielded an estimated power of 100%, indicating that the study was sufficiently powered to detect the observed effect.

### 2.4. Study Participants and Sampling Criteria

We used a consecutive sampling method to recruit caregivers of children under 18 years who attended SCD clinics at Temeke RRH, Amana RRH, MNH-Upanga, and MNH-Mloganzila hospitals. All caregivers had children with health insurance and therefore free access to HU [43]. We focused on caregivers because more than three-quarters of patients with SCD in Tanzania, as reported in the SPARCO registry, are children under 18 years [17,43] and thus still under care, and as a result, their caregivers likely influence their HU utilization. All patients included in this study had a confirmed SCD diagnosis by hemoglobin electrophoresis or high-performance liquid chromatography [43]. All caregivers enrolled in the study were caring for children with SCD who were registered in the SPARCO database. This allowed us to confirm the diagnosis of SCD, the genotype of SCD, and self-reported status of HU use among children with SCD. By the year 2023, 65% of children with SCD attending the study sites were health-insured, and most of the insured children had a National Health Insurance Fund (NHIF) subscription, as reported in the SPARCO database [17]. There are no restrictions on HU coverage among the NHIF beneficiaries in Tanzania [4]. The exclusion of caregivers with uninsured children with SCD allowed us to study the effect of self-efficacy on HU use while controlling for the cost of HU, which is the major barrier to HU use in Tanzania [42].

### 2.5. Data Collection Tools

We adapted the standardized data collection tools validated in other studies. The tools were translated into Swahili using a standardized forward and backward translation process. We also conducted a pilot test among a small group of caregivers (*n* = 12) to assess the clarity and cultural appropriateness of the items. We made minor modifications based on the feedback to improve comprehension and adapted them to local circumstances without altering the core constructs. We calculated the reliability of the adapted tools after data collection by assessing the internal consistency of the scales using Cronbach’s alpha. All scales had reliabilities of Cronbach’s alpha of 0.9. The original validated tools are available online [44,45,46].

We assessed self-efficacy by using the 13-item Self-Efficacy for Appropriate Medication use Scale (SEAMS). SEAMS has a score range of 13–39. The scale assesses the perceived individual’s confidence of using medication under difficult circumstances and when the circumstances surrounding medication-taking are uncertain. The higher the score, the higher the perceived self-efficacy. Participants with a SEAMS score of less than 26 (67%) were considered to have low self-efficacy, whereas those with scores of 26 and above were categorized as having high perceived self-efficacy [44]. SEAMS has been validated in previous studies and found to have reliability of Cronbach’s alpha of 0.9 [20,21,22,47]

Social support was assessed by using the 12-item Multidimensional Perceived Social Support Scale (MPSSS). The scale was developed by Gregory Zimet et al., and it assesses an individual’s perceived social support from significant others, family, and friends. The highest score for the MPSSS is 7, which is obtained by calculating the mean of the total score. A score less than 3 is considered low support, 3–5 is considered moderate support, and above 5 is considered high support [48]. The reliability of MPSSS obtained from other studies ranges from a Cronbach’s alpha of 0.8 to 0.9 [49,50]

Depressive symptoms (at least within the past two weeks prior to enrolment in the study) were assessed by using the 2-item Patient Health Questionnaire (PHQ-2), which is a subset of the PHQ-9 scale. The PHQ-2 scale has a total score of 6. Participants with scores less than 3 were considered as not having depressive symptoms, whereas those with a score of 3 and above were considered to have depressive symptoms [46]. The PHQ-2 scale has been validated in previous studies and found to have a reliability of Cronbach’s alpha of 0.9 [46,51].

### 2.6. Ethical Approvals

We obtained ethical approval from the institutional review boards at Muhimbili University of Health and Allied Sciences (MUHAS-REC-04-2023-1640) and National Institution for Medical Research (NIMR/HQ/R.8a/Vol.IX/4409). All caregivers provided written informed consent prior to their enrolment in the study.

### 2.7. Data Analysis

We performed all analyses using the Statistical Package for Social Scientists (SPSS v29.0.1.0). In our study, the dependent variable was caregivers’ perceived self-efficacy for appropriate use of HU. We summarized sociodemographic characteristics, depressive symptoms, and social support as frequencies and proportions. We used the Shapiro–Wilk test to assess the distribution of perceived self-efficacy scores. The scores were not normally distributed and were hence summarized as median (Inter-Quartile Range, IQR) scores. To ascertain the association between different caregivers’ characteristics and median scores for self-efficacy, we used the Mann–Whitney U test for binary categories and the Kruskal–Wallis test for variables with three categories. The scores were also dichotomized into low and high scores to obtain the prevalence of high self-efficacy among participants and facilitate interpretation. We used quantile regression for the analysis of factors associated with perceived self-efficacy. From the existing literature, sociodemographic and psychosocial variables affect self-efficacy; therefore, we included all variables in the multivariable analysis, and the factors were adjusted for all other factors. However, there was a significant correlation between caregivers’ education and occupation, indicating collinearity with a *p*-value of <0.001. Therefore, we excluded occupation from the multivariate analysis. We analyzed the association between caregivers’ perceived self-efficacy for appropriate use of HU and the actual HU use among children with SCD using the modified Poisson regression. The differences in comparisons with a *p*-value of <0.05 were considered statistically significant.

## 3. Results

### 3.1. Sociodemographic Characteristics of Study Participants

We enrolled 374 caregivers of children with SCD. Women contributed 86% of all caregivers. Sixty-five percent of caregivers were young adults below the age of 45 years. Fifty-four percent had secondary or higher education. Seventy-two percent were either formally employed or self-employed. Most caregivers (80%) had children with SCD aged 12 years or younger. Thirty-seven percent were enrolled at the regional hospitals. Sixteen percent of caregivers reported to have experienced depressive symptoms over the last 2 weeks prior to enrolment. Thirty-two percent perceived having low-to-moderate social support from their family, friends, or significant others. Fifty-six percent of caregivers at national hospitals had secondary or higher education, whereas fifty-four percent of those at regional hospitals had only primary education or no formal education. However, there was no statistically significant difference in education level or employment status among caregivers of children enrolled at regional-level versus national-level hospitals (Table 1).

### 3.2. Factors Associated with Caregivers’ Perceived Self-Efficacy for Appropriate Use of Hydroxyurea

The median (IQR) score for self-efficacy was 34 (25–37). The lowest score was 13/39, whereas the highest score was 39/39. Seventy-five percent of caregivers had high self-efficacy scores of ≥26. High self-efficacy scores were more often observed among caregivers attending clinics at national hospitals compared to those attending regional hospitals {35 (31, 38) vs. 28 (20, 36), *p* = 0.001}. We also observed that high self-efficacy scores were higher among those with high perceived social support compared to those with low-to-moderate social support {35 (31, 38) vs. 23 (20, 34), *p* ≤ 0.001}. Further, high self-efficacy scores were higher among caregivers who did not have depressive symptoms compared to those with depressive symptoms {35 (29, 38) vs. 22 (18, 33), *p* = 0.001} (Table 1).

Upon analysis within variables, we observed that the proportion of caregivers with high perceived social support was higher among caregivers attending national hospitals than those attending regional hospitals (79% vs. 49%, *p* = 0.001). We noted that depressive symptoms were more prevalent among caregivers attending regional hospitals than those at national hospitals (30% vs. 7%, *p* = 0.001). Further, depressive symptoms were more observed among caregivers with low-to-moderate perceived social support than in caregivers with high perceived social support (38% vs. 5%, *p* = 0.001).

Upon multivariable analysis, caregivers attending the national hospitals were 2.3 times more likely to have high self-efficacy than those attending regional hospitals (RRHs) (aβ 2.3, 95% CI 0.5–4.2). Caregivers with high social support were 9 times more likely to have high self-efficacy than those with low-to-moderate support (aβ 9, 95% CI 7.1–10.9). Caregivers who had no depressive symptoms were 5.3 times more likely to have high self-efficacy compared to those with depressive symptoms (aβ 5.3, 95% CI 2.8–7.8) (Table 2).

### 3.3. Association Between Caregivers’ Perceived Self-Efficacy and HU Use Among Children with SCD

Seventy percent of the caregivers reported giving HU to their children with SCD. Eighty-seven percent of the caregivers with high perceived self-efficacy reported giving HU to their children, whereas only fifteen percent of children of caregivers with low perceived self-efficacy were using HU. Caregivers with high perceived self-efficacy for appropriate use of HU were 5.3 times more likely to give HU to their children compared to those with low perceived self-efficacy (IRR 5.3, 95% CI 3.3–8.3) (Table 3).

## 4. Discussion

Understanding the factors associated with self-efficacy for appropriate use of HU is crucial for improving the use of HU and patients’ outcomes. This study provides the first evidence from Tanzania showing that a lack of social support, exhibiting depressive symptoms, and receiving care at lower-level healthcare facilities were associated with caregivers’ low perceived self-efficacy for appropriate use of HU and subsequently lower HU utilization among children with SCD. These findings highlight the need to scale-up public health interventions aimed at enhancing social and psychological support to caregivers in order improve their perceived self-efficacy and consequently increase HU usage among children with SCD in Tanzania.

We observed that one-third of caregivers had low-to-moderate social support from their significant others, families, and friends. Caregivers with greater perceived social support were nine times more likely to have high perceived self-efficacy for appropriate use of HU compared to those with low and moderate social support. This is similar to findings from other studies showing that social support improves self-efficacy, self-management, and health-related quality of life [52,53,54]. Supportive interactions with family, friends, and others offer emotional encouragement and motivation to use medications, provide access to health information, and assist in organizing medications and setting reminders on when to use the prescribed medications [26,27,28]. Social support may also buffer some of the socioeconomic constraints by helping caregivers navigate health systems, provide transportation, or share caregiving responsibilities. This strengthens an individual’s confidence and ability to use medications effectively. In contrast, caregivers with limited social support often face barriers to healthcare, such as transport challenges and lost income from missed work [33]. These barriers disrupt access to care and contribute to emotional exhaustion, perception that outcomes are out of one’s control, reduced self-efficacy, and eventually reduced motivation to engage in health behaviors [33]. Therefore, social support, self-efficacy, and structural factors are important constructs in optimizing the use of prescribed medicines in patients with chronic diseases such as SCD [55,56]. We recommend engagement in facilitated SCD support groups to improve perceived support and reducing feelings of hopelessness among patients and caregivers of patients with SCD [57].

We also observed that 16% of caregivers reported exhibiting depressive symptoms. Studies have shown that SCD is associated with high psychosocial burden among patients with SCD and their families [1,4,10,58]. In our study, we observed that a lack of depressive symptoms among caregivers was associated with high self-efficacy for appropriate use of HU. This is similar to findings from other studies showing that depressive symptoms are negatively associated with self-efficacy, whereas positive emotional well-being influences medication usage [59,60]. Depression may lead to negative health beliefs and perceptions, impair motivation towards medications, and subsequently diminish self-efficacy [27,34,60]. Therefore, the assessment and management of depressive symptoms among caregivers of children with SCD is vital in improving self-efficacy and HU use among children with SCD. In our study, we used the PHQ-2 scale as a brief and feasible tool to screen for depressive symptoms among caregivers of children with SCD attending in a busy, resource-constrained clinic setting [51]. We selected the PHQ-2 scale due to its conciseness and ease of administration, considering the time and staffing constraints that limit the use of longer screening tools such as the PHQ-9 scale. However, we acknowledge that it may not fully capture the severity of depressive symptoms as well as the PHQ-9 would do. Therefore, we recommend that, where possible, caregivers who screen positive on the PHQ-2 be followed-up using the PHQ-9 scale and further clinical assessment to improve the identification and management of individuals with depressive symptoms [46,51].

Further, we found that caregivers attending SCD clinics at national hospitals were 2.3 times more likely to have high perceived self-efficacy compared to those attending regional hospitals. Several factors may contribute to this observation. Firstly, an earlier study in the same setting reported that caregivers attending regional hospitals had inadequate knowledge of SCD and lower scores of perceived threats of SCD complications compared to those attending national hospitals [43]. It should be noted that although we did not find significant differences in caregiver education level across hospitals, the majority of caregivers at national hospitals had secondary or higher education, whereas the majority of those at regional hospitals had primary education or less. Studies have shown that a high level of education is associated with greater health literacy, stronger communication with healthcare providers, and better understanding of disease management strategies, which are crucial in improving the attitude and skills in medication-taking behaviors [61,62]. Despite the lack of significant differences in education levels among caregivers across the study sites, caregivers at national hospitals may still have greater SCD knowledge and health literacy than those at regional hospitals. Other studies have reported that it is possible for individuals with similar levels of formal education to have varying degrees of health literacy [63]. Additionally, nurses who often provide health education to patients and caregivers in regional hospitals were found to have suboptimal knowledge on the management of SCD [43,64]. At national hospitals, health education is often provided by specialists and hematology trainees who have a higher knowledge of SCD management than providers offering health education sessions at regional hospitals [43]. This limits the SCD health information provided to caregivers and further limits caregivers’ health literacy. Therefore, although we did not directly assess caregivers’ health literacy and SCD knowledge, we can hypothesize that caregivers attending national hospitals had a higher level of health literacy compared to those at regional hospitals. Implementing standardized caregiver education programs, alongside regular staff training, can help ensure consistent delivery of essential information on SCD management. Secondly, the proportion of caregivers with high perceived social support was higher among caregivers attending national hospitals compared to those attending regional hospitals, whereas depressive symptoms were more prevalent among caregivers attending regional hospitals than those at national hospitals. This may have been caused by the differences in the availability of social welfare and mental health services between the regional and national hospitals [65]. There are ongoing efforts to improve mental health in Tanzania, such as the integration of mental health into primary care and community health services and the coverage of mental health care services in the NHIF, the inclusion of mental health in national policies, budget allocation, and training activities. However, mental health services are still under-resourced, and this limits caregivers’ access to mental health care and further reduces self-efficacy [10,65]. Thirdly, the higher perceived self-efficacy scores observed among caregivers at national hospitals may have been caused by differences in disease severity among children with SCD across the sites. Caregivers of patients with severe diseases tend to have better understanding of diseases and positive beliefs towards medications, which influence self-efficacy and medication usage [43]. MNH is the highest referral hospital in Tanzania, and it therefore receives patients with the most severe SCD complications compared with those at regional hospitals. However, we acknowledge that all caregivers enrolled in our study had children with the most severe SCD genotype (HbSS), and therefore, objective assessment of disease severity is needed to strengthen this observation. Overall, the differences in caregivers’ perceived self-efficacy for the appropriate use of HU may have been caused by the differences in the quantity and quality of knowledge, health information, and counseling services provided to caregivers of children with SCD at these facilities. We recommend future studies to incorporate indicators for the assessment of caregivers’ health literacy, SCD severity, and quality of SCD education provided at these hospitals.

Lastly, we noted that high self-efficacy was positively associated with HU use among children with SCD. This is similar to findings from other studies showing that high medication self-efficacy is associated with optimal use of medications and self-management [23,66,67,68]. When individuals believe that they have appropriate skills to mitigate a particular risk, they are more likely to take the appropriate measures to prevent it [21]. However, a few studies have shown no correlation between self-efficacy and optimal medication use [69,70]. It is speculated that people with high medication self-efficacy might simply decide not to take the prescribed medications regularly [68]. To enhance medication use, more efforts should be directed to improve self-efficacy on medication use among patients and caregivers of patients with chronic diseases such as SCD. Our assessment relied on self-reported HU use, which is subject to social desirability bias and may overestimate the actual use of HU. To strengthen accuracy, we confirmed the self-reported HU use by checking the child’s HU status reported in the SPARCO database. We recommend the incorporation of objective measures such as HU pharmacy refill data or assessment of fetal hemoglobin to enhance the accuracy of HU use assessment in similar settings.

A major strength of this study was the enrolment of a large number of caregivers of children with SCD attending at different levels of healthcare facilities. The inclusion of caregivers of health-insured patients attending clinics in urban hospitals may have led to an overestimation of self-efficacy and HU use. However, studying the selected participants controlled the effects of the major barriers to HU use in Tanzania, i.e., unavailability and unaffordability of HU, and it therefore allowed us to understand the actual effect of perceived self-efficacy on HU use. This cements the significance of incorporating targeted interventions to improve caregiver-related factors together with ongoing interventions to improve health-system-related factors in Tanzania. Additionally, we evaluated caregivers’ perceived social support and depression, shedding light on the psychosocial burden faced by families of children with SCD. Limitations of this study include the use of consecutive sampling, which may have introduced selection bias by potentially excluding caregivers with irregular clinic attendance who may face greater challenges related to perceived self-efficacy and hydroxyurea usage. Other limitations are the inability to explore caregivers’ levels of health literacy, the effect of cultural influences, and the number of children with SCD or other chronic diseases in the families, which could further explain their perceived self-efficacy by either increasing SCD knowledge or adding psychosocial burden to caregivers. To provide a more comprehensive understanding of factors affecting self-efficacy for HU use, future studies should employ broader recruitment strategies, including outreach to caregivers with inconsistent follow-up, incorporate assessment of caregivers’ health literacy, and inquire about cultural influences and the number of children with chronic diseases in the family.

## 5. Conclusions

Three-quarters of caregivers of children with SCD attending clinics in public secondary and tertiary health facilities reported high perceived self-efficacy for appropriate use of HU. We found that greater social support and attending a higher-level health facility were positively associated with self-efficacy, whereas the presence of depressive symptoms had a negative association. Further, caregivers’ perceived self-efficacy for appropriate HU use influenced the use of HU among children with SCD. We recommend integrating public health interventions into the ongoing efforts to increase HU use in Tanzania. These programs should address the psychosocial needs of caregivers, such as structured peer education programs where experienced caregivers share knowledge about SCD management and coping strategies. Further, encouraging engagement in SCD support groups can provide emotional support and reduce caregiver isolation and depressive symptoms. Moreover, improving access to mental health services through integration of mental health care in dedicated SCD clinics may strengthen the psychological well-being of caregivers and patients with SCD and subsequently improve caregivers’ perceived self-efficacy and increase HU usage among children with SCD in Tanzania. Future studies should evaluate the effectiveness of these interventions in similar settings.

## Figures and Tables

**Table 1:** Sociodemographic characteristics of the study participants.

Variable		Frequency (%)	Perceived Self-Efficacy		*p*-Value
			Median Scores (IQR)	Test Used	
Caregivers’ sex	Male	54 (14)	33 (25, 37)	Mann–Whitney U test	0.065
	Female	320 (86)	34 (25, 37)		
Caregivers’ age (years)	<35	102 (27)	34 (26, 38)	Kruskal–Wallis test	0.394
	35–44	142 (38)	34 (23, 37)		
	≥45	130 (35)	35 (23, 37)		
Caregivers’ education level	No formal education and primary education	173 (46)	34 (23, 37)	Mann–Whitney U test	0.154
	Secondary and higher education	201 (54)	35 (27, 37)		
Caregivers’ occupation	Not employed	315 (84)	34 (28, 37)	Mann–Whitney U test	0.478
	Employed	269 (72)	34 (23, 37)		
Child’s age group (years)	<5	76 (20)	34 (24, 38)	Kruskal–Wallis test	0.178
	5–12	224 (60)	34 (23, 37)		
	13–17	74 (20)	35 (28, 38)		
Level of health facility	Regional	138 (37)	28 (20, 36)	Mann–Whitney U test	0.001
	National	236 (63)	35 (31, 38)		
Depressive symptoms	No	314 (84)	35 (29, 38)	Mann–Whitney U test	0.001
	Yes	60 (16)	22 (18, 33)		
Social support	Low to Moderate	120 (32)	23 (20, 34)	Mann–Whitney U test	<0.001
	High	254 (68)	35 (31, 38)		

**Table 2:** Factors associated with high perceived self-efficacy for appropriate use of HU.

Variable		Median Scores (IQR)	Univariable Analysis		Multivariable Analysis	
			cβ	95% CI	aβ	95% CI
Caregivers’ sex	Female	34(25, 37)	1	−1.8–3.8	0.7	−1.7–3.1
	Male	33.5(25, 37)	Ref			
Caregivers’ age	<35	34(26, 38)	Ref			
	35–44	34(23, 37)	0	−2.7–2.6	−2	−4.2–0.2
	≥45	35(23, 37)	1	−1.7–3.7	−1.3	−3.6–0.9
Caregivers’ education level	No formal education and primary education	34(23, 37)	Ref			
	Secondary and higher education	35(27, 37)	1	−1.1–3.1	0.7	−1.1–2.3
Child’s age group (years)	<5	34(25, 38)	Ref			
	5–12	34(23, 37)	0	−2.8–2.8	−0.7	−2.8–1.4
	13–17	35(28, 38)	1	−2.4–4.4	0.3	−2.4–3.1
Level of health facility	Regional	28(20, 36)	Ref			
	National	35(31, 38)	7	4.7–9.3	2.3	0.5–4.2
Depressive symptoms	No	35(29, 38)	13	10.7–15.3	5.3	2.8–7.8
	Yes	22(18, 33)	Ref			
Social support	Low to Moderate	23(20, 34)	Ref			
	High	35(31, 38)	16	14.8–17.1	9	7.1–10.9

Key: cβ: crude beta coefficient, aβ: adjusted beta coefficient, Ref: reference category, CI: confidence interval. The factors were adjusted for all other variables shown in the table.

**Table 3:** Association between caregivers’ perceived self-efficacy and hydroxyurea use among children with SCD.

Variable	Category	Hydroxyurea Use		IRR	95% CI
		Yes (%)	No (%)		
Perceived self-efficacy	High (%)	248 (87)	36 (13)	5.3	3.3–8.3
	Low (%)	15 (17)	75 (83)	Ref	

Key: IRR: incidence rate ratio, Ref: reference category, CI: confidence interval.

## Data Availability

The raw data supporting the conclusions of this study will be made available by the corresponding author on request.
